# Effects of omeprazole on symptoms and quality of life in Japanese patients with reflux esophagitis: Final results of OMAREE, a large-scale clinical experience investigation

**DOI:** 10.1186/1471-230X-11-15

**Published:** 2011-02-28

**Authors:** Shigeru Yoshida, Masahiro Nii, Masataka Date

**Affiliations:** 1 Research & Development, AstraZeneca K.K. 1-1-88 Ohyodo-naka, Kita-ku, Osaka 531-0076, Japan

## Abstract

**Background:**

For patients with reflux esophagitis (RE), endoscopic findings alone (without the frequency and severity of symptoms) may not fully reflect the associated impact on health-related quality of life (QOL). There is not enough data about symptoms and QOL of Japanese patients with RE. The present study therefore investigated the epidemiological characteristics of such patients, and evaluated the efficacy and safety of omeprazole (and other gastrointestinal drugs, except proton pump inhibitors [PPIs]) in terms of improving patients' symptoms and QOL.

**Methods:**

In a large-scale, specific clinical experience investigation of Japanese patients with RE, epidemiological characteristics, QOL and symptoms of the disease in relation to treatment with omeprazole and other gastrointestinal drugs, except PPIs, and safety data of omeprazole were collected. The Quality Of Life in Reflux and Dyspepsia questionnaire (QOLRAD) was used for QOL assessment.

**Results:**

9967 patients were included in the analysis (omeprazole: 7888). At baseline, 75.2% of patients had three or more upper gastrointestinal symptoms, and 31.5% of patients had six or more upper gastrointestinal symptoms. The overall mean QOLRAD score at baseline was 5.14 (the best score is 7). In the omeprazole group, the rate of satisfactory improvement in subjective symptoms was 61.7% and 81.8% at Weeks 4 and 8, respectively, and these were both significantly higher than those of patients treated with other drugs. In both the omeprazole group and the other drugs group, the QOLRAD score at Week 4 improved significantly from baseline, and the degree of improvement was significantly greater in the omeprazole group than in the other drugs group. The favourable tolerability profile of omeprazole was confirmed.

**Conclusion:**

In a large-scale survey, omeprazole improved symptoms and QOL more effectively in Japanese patients with RE than other investigated drugs, and had a good tolerability profile.

**Trial Registration:**

ClinicalTrials.gov identifier: NCT00859287.

## Background

In the evaluation of clinical efficacy, subjective outcomes are now being regarded as very important, and patient-based clinical outcome measures are being required in addition to conventional indices of pathological changes. For reflux esophagitis (RE) in particular, some reports have shown that endoscopic findings do not correspond to the frequency and severity of symptoms[1] and that the health-related quality of life (QOL) of patients with RE is as impaired as that of patients with angina pectoris[2]. Elsewhere, in recent studies in Japan, the frequency of symptoms and the severity of endoscopic findings were associated[3,4]. Nonetheless, assessing QOL is an important facet of evaluating clinical treatments.

Various scales and questionnaires have been developed to assess QOL, including the MOS Short-form Health Survey (SF-36)[5] as a health profile scale, the Psychological General Well-Being scale (PGWB)[6] to measure psychosocial factors, the Gastrointestinal Symptom Rating Scale (GSRS)[7] to evaluate general gastrointestinal symptoms, and the Quality Of Life in Reflux And Dyspepsia (QOLRAD) questionnaire[8] as a disease-specific questionnaire for RE.

QOLRAD-J, the Japanese version of QOLRAD, was recently developed and validated for assessment of QOL in Japanese patients with heartburn[9]. The QOLRAD-J consists of 25 questions grouped into five domains (emotional distress, sleep disturbance, food/drink problems, physical/social functioning, and vitality) that are strongly related to acid reflux symptoms. Each domain is scored using a scale from 1 (worst condition) to 7 (best condition). A quantitative psychological evaluation of the QOLRAD-J in Japanese patients with heartburn demonstrated sufficient reliability, validity and distinctiveness.

It has been described that the prevalence of upper abdominal symptoms such as heartburn is high and QOL is significantly impaired in patients with gastroesophageal reflux disease (GERD), including RE[10,11]. Nevertheless, no large-scale epidemiological survey has been conducted in Japan to assess the symptoms or QOL of patients with heartburn or other syndromes, or the relationship between patient characteristics and treatment. Thus, we used the QOLRAD-J and a questionnaire to record clinical symptoms and evaluate the efficacy and safety of omeprazole in the treatment of RE. This report describes the QOL and symptoms of Japanese patients with RE, as well as changes in their QOL and symptoms after treatment with omeprazole or other gastrointestinal drugs, except for proton pump inhibitors (PPIs), and the safety profile of omeprazole.

## Methods

### Objectives

The objectives of this investigation were to epidemiologically analyze the background factors, clinical symptoms and QOL of patients with RE using the QOLRAD-J questionnaire as part of usual clinical practice in Japan, and to evaluate the effects of omeprazole on their symptoms and QOL; the safety and tolerability of omeprazole was also determined. Other non-PPI gastrointestinal drugs (subsequently referred to as 'other drugs'; see details below) were also evaluated in terms of their effects on symptoms and QOL of RE patients. This was a specific investigation of clinical experience entitled OMAREE (Omepral^® ^tablets Mega-study to investigate the efficacy on various types of Acid Reflux related symptoms and QOL, and epidemiology in patients with Erosive Esophagitis in daily medical practice) and was conducted in compliance with Good Postmarketing Study Practice and Helsinki Declaration, and in accordance with the Personal Information Protection Law. The institutional review board at Jinbo Orthopedics Clinic and the join institutional review board at Keihin-Chuo Clinic, Hisamitsu Clinic, and Masabayashi Clinic approved the study design (Reference Study Number: D9584L00008). Study objectives and design were explained and consent was obtained from all patients. This study is registered with ClinicalTrials.gov, number NCT00859287.

### Patients

To be included in the investigation, patients had to be receiving (either as initial therapy or maintenance therapy for RE) or be newly started on omeprazole (Omepral^® ^Tablet 10 or Omepral^® ^Tablet 20, AstraZeneca K.K., Japan) or other drugs but not within 4 weeks of study initiation, have had acid reflux-related symptoms (e.g., heartburn) of any intensity for 2 days or more, or of moderate to severe intensity for 1 day or more during 1 week immediately before the start of the investigation, and be able to complete the questionnaires to record symptoms and QOLRAD-J. Endoscopic confirmation of the diagnosis of RE was also required. Patients were excluded if they had received PPI therapy within 4 weeks before the start of the investigation, had been previously enrolled in this investigation, or were contraindicated to treatment with omeprazole according to the package insert. During the investigation, all treatments were provided at the physician's discretion and in an open-label manner. This investigation included also special patient populations such as children, elderly patients, patients with renal dysfunction, patients with hepatic dysfunction, or pregnant women.

### Methods and outcome variables

The enrollment period of this investigation was between June 2007 and May 2008, and patients were followed up for 8 weeks. Five patients were enrolled per center.To avoid an imbalance of patient characteristics between the treatment groups, the patient who had been or would be treated with other drugs was enrolled as the first patient at each participating center, while the patients who had been or would be treated with omeprazole were enrolled as the second to fifth patients.

On enrollment, the patient's age, sex, height, body weight, lifestyle, medical history, surgical history, prior medication, *Helicobacter pylori *infection status, history of the present illness, and QOL (measured using the QOLRAD-J) were recorded. Symptoms were assessed by both the investigators (i.e., physician-reported symptoms) and the patients (i.e., patient-reported symptoms). The investigators recorded the chief complaint of the patient, and its severity and frequency. The patients recorded the type, severity and frequency of the symptoms, the chief complaint, and the area affected, directly on the questionnaire form. Physician- and patient-reported symptoms, and QOL were to recorded at Weeks 4 and 8 of the investigation, based on recall relating to the previous week. Patients in the omeprazole group were also monitored for any adverse drug reactions (drug-related adverse events [as assessed by the physician]) during the investigation period.

### Statistical methods

The final analysis was conducted using patients for whom data collection and data fixation had been completed as of March 31, 2009.

Patient background characteristics and clinical symptoms at baseline were summarized and descriptive statistics were calculated. For QOL, the QOLRAD-J scores were calculated and, to verify the validity of the QOLRAD-J, Cronbach's α coefficients for individual domains and QOLRAD-J scores stratified by the severity and frequency of physician-reported symptoms were calculated. For each patient, if 50% or more of the items within a specific domain of the QOLRAD-J were answered, the mean score of the answered items was used as the domain score; if less than 50% of the items were answered within the domain, the domain score was regarded as missing.

The primary endpoint for the efficacy assessment was a satisfactory improvement in subjective symptoms at Week 8, defined as physician-reported symptoms during the preceding week being "None" or "Only mild symptoms for 1 day". This definition was used throughout the study. The secondary endpoints included a satisfactory improvement in subjective symptoms at Week 4, complete resolution of subjective symptoms (i.e., physician-reported symptoms during the preceding week being "None") at Weeks 4 and 8, satisfactory improvement in individual patient-reported symptoms at Weeks 4 and 8, and QOLRAD scores before and after the start of the investigation with stratification for physician-reported symptom. The primary and secondary endpoints were compared between the groups, while also compared between before and after the start of the investigation.

Between-group comparisons of the rates of improvement and resolution of symptoms were conducted using χ^2 ^tests. Between-group comparisons of QOLRAD score were conducted using two-sample *t *tests, and the change from baseline in the QOLRAD score within each treatment group was analyzed using paired *t *tests without multiplicity adjustment. A two-sided 5% significance level was used for all statistical tests. For the evaluation of safety and tolerability, the incidence of adverse drug reactions in the omeprazole group was analyzed descriptively.

In this study, enrolled patients will be divided into two groups (omeprazole: other drugs = 4:1). The efficacy rates of the primary endpoint were assumed to be 50% and 45%, respectively, for the two groups. In order to have 95% power to detect a difference in the efficacy rates between the groups using a χ^2 ^test with two-sided 5% significance level, 6472 omeprazole-treated patients and 1618 patients for 'other drugs' was required. So, it was necessary to enroll a total of at least 8090 patients.

## Results

### Patient characteristics

A total of 2143 medical institutions participated in this investigation, and data were collected for 10,704 patients. The analysis set consisted of 9967 patients (7888 patients received omeprazole and 2079 patients received other drugs); 737 patients were excluded from the analysis because of inappropriate enrollment, inability to collect data or contract violation. The safety analysis set consisted of 7711 patients treated with omeprazole, as 177 patients did not return to the investigation site after enrollment and were excluded.

Table 1 shows the characteristics of the patients in the analysis set. Overall, there was a slightly larger number of women than men. The mean body mass index was 23.23 kg/m^2^, showing no tendency for obesity. Concurrent conditions reported in ≥5% of patients included hypertension (28.7%), hyperlipidemia (18.4%), chronic or acute gastritis (15.4%), esophageal hiatus hernia (8.5%), insomnia (7.6%), and diabetes mellitus (5.9%).

**Table 1:** Demographic and baseline characteristics

		Total (N = 9967)	Omeprazole (N = 7888)	Other drugs (N = 2079)
Age	< 40 years	1503 (15.1)	1169 (14.8)	334 (16.1)
	≥40 - <65 years	3929 (39.4)	3107 (39.4)	822 (39.5)
	≥65 years	4535 (45.5)	3612 (45.8)	923 (44.4)
Sex	Men	4178 (41.9)	3246 (41.2)	932 (44.8)
	Women	5789 (58.1)	4642 (58.9)	1147 (55.2)
Body mass index, kg/m^2^	< 18.5	404 (4.1)	301 (3.8)	103 (5.0)
	≥18.5 - <25	4134 (41.5)	3234 (41.0)	900 (43.3)
	≥25	1786 (17.9)	1378 (17.5)	408 (19.6)
	Unknown	3643 (36.6)	2975 (37.7)	668 (32.1)
Habitual smoking	No	7310 (73.3)	5744 (72.8)	1566 (75.3)
	Yes	1685 (16.9)	1333 (16.9)	352 (16.9)
	Unknown/not specified	972 (9.8)	811 (10.3)	161 (7.7)
Habitual alcohol consumption	No	5777 (58.0)	4608 (58.4)	1169 (56.2)
	Yes	3180 (31.9)	2444 (31.0)	736 (35.4)
	Unknown/not specified	1010 (10.1)	836 (10.6)	174 (8.4)
*H. pylori *eradication	No	5712 (57.3)	4503 (57.1)	1209 (58.2)
	Yes	583 (5.9)	454 (5.8)	129 (6.2)
	Unknown	3672 (36.8)	2931 (37.2)	741 (35.6)
*H. pylori *infection	No	3003 (30.1)	2348 (29.8)	655 (31.5)
	Yes	513 (5.2)	415 (5.3)	98 (4.71)
	Unknown/not specified	6451 (64.7)	5125 (65.0)	1326 (63.8)

**Past and concurrent diseases (reported in ≥5% patients)**				
Past disease	No	8480 (85.1)	6763 (85.7)	1717 (82.6)
	Yes	1487 (14.9)	1125 (14.3)	362 (17.4)
Concurrent disease	No	3789 (38.0)	2940 (37.3)	849 (40.8)
	Yes	6178 (62.0)	4948 (62.7)	1230 (59.2)
Esophageal hiatus hernia	Past disease	26 (0.3)	19 (0.2)	7 (0.3)
	Concurrent disease	844 (8.5)	690 (8.8)	154 (7.4)
Gastritis (chronic/acute)	Past disease	258 (2.6)	190 (2.4)	68 (3.3)
	Concurrent disease	1536 (15.4)	1278 (16.2)	258 (12.4)
Diabetes mellitus	Past disease	45 (0.5)	32 (0.4)	13 (0.6)
	Concurrent disease	586 (5.9)	463 (5.9)	123 (5.9)
Hyperlipidaemia	Past disease	129 (1.3)	98 (1.2)	31 (1.5)
	Concurrent disease	1831 (18.4)	1477 (18.7)	354 (17.0)
Hypertension	Past disease	151 (1.5)	120 (1.5)	31 (1.5)
	Concurrent disease	2860 (28.7)	2290 (29.0)	570 (27.4)
Insomnia	Past disease	48 (0.5)	36 (0.5)	12 (0.6)
	Concurrent disease	756 (7.6)	592 (7.5)	164 (7.9)

**Duration of RE**				
	< 3 months	6236 (62.6)	5041 (63.9)	1195 (57.5)
	≥3 - <6 months	963 (9.7)	776 (9.8)	187 (9.0)
	≥6 months	2767 (27.8)	2070 (26.2)	697 (33.5)
	Not specified	1 (0)	1 (0)	0 (0)

**Prior treatment for RE**				
Treatment for RE during the 4 weeks prior to the investigation	No	7769 (78.0)	6218 (78.8)	1551 (74.6)
	Yes	2007 (20.1)	1532 (19.4)	475 (22.9)
	H_2 _blockers	1519 (15.2)	1161 (14.7)	358 (17.2)
	Prokinetics	509 (5.1)	391 (5.0)	118 (5.7)
	Antacids	197 (2.0)	151 (1.9)	46 (2.2)
	Mucosal protectants	536 (5.4)	410 (5.2)	126 (6.1)
	Antianxiety drugs	101 (1.0)	69 (0.9)	32 (1.5)
	Antidepressants	47 (0.5)	32 (0.4)	15 (0.7)
	Chinese herbal medicines	59 (0.6)	37 (0.5)	22 (1.1)
	Other drugs	95 (1.0)	71 (0.9)	24 (1.2)
	Unknown	191 (1.9)	138 (1.8)	53 (2.6)
PPI use during the past 5 years				
	No	6953 (69.8)	5462 (69.2)	1491 (71.7)
	Yes	1761 (17.7)	1444 (18.3)	317 (15.3)
	< 6 months	809 (8.1)	663 (8.4)	146 (7.0)
	≥6 months - <1 year	408 (4.1)	337 (4.3)	71 (3.4)
	≥1 - <2 years	323 (3.2)	259 (3.3)	64 (3.1)
	≥2 - <5 years	213 (2.1)	178 (2.3)	35 (1.7)
	≥5 years	8 (0.1)	7 (0.1)	1 (0.1)
	Unknown	1253 (12.6)	982 (12.5)	271 (13.0)

Values are n (%). RE = reflux esophagitis; PPI = proton pump inhibitor.

The duration of RE was reported as less than 3 months in 62.6% of patients, and was 6 months or more in 27.8% of patients. In terms of the drugs used during the 4 weeks before the start of the investigation (patients using PPIs during this time were excluded), H_2 _blockers were the most commonly used (15.2%). When we investigated the previous 5 years, 17.7% of patients had a history of PPI therapy.

In patients treated with other drugs, the most commonly used agents were H_2 _blockers (1895 patients, 91.2%), prokinetics (490 patients, 23.6%), and mucosal protectants (435 patients, 20.9%).

### Clinical symptoms and QOL at baseline

Table 2 shows the frequency and severity of physician-reported symptoms at baseline. Moderate or severe symptoms were reported for 57.5% of patients. The ratio of patients with moderate or severe symptoms in the omeprazole group was slightly higher than in the other drugs group. 41.4% of patients were symptomatic almost every day.

**Table 2:** Physician-reported symptoms at baseline

		Total (N = 9967)	Omeprazole (N = 7888)	Other drugs (N = 2079)
**Intensity (%)**				
	Mild	4235 (42.5)	3127 (39.6)	1108 (53.3)
	Moderate	5273 (52.9)	4351 (55.2)	922 (44.4)
	Severe	459 (4.6)	410 (5.2)	49 (2.4)
**Frequency (%)**				
	Not specified	14 (0.1)	10 (0.1)	4 (0.2)
	1 day	161 (1.6)	124 (1.6)	37 (1.8)
	2 days	1348 (13.5)	993 (12.6)	355 (17.1)
	3 days	1964 (19.7)	1528 (19.4)	436 (21.0)
	4 days	1124 (11.3)	892 (11.3)	232 (11.2)
	5 days	1230 (12.3)	984 (12.5)	246 (11.8)
	6 days	327 (3.3)	271 (3.4)	56 (2.7)
	7 days	3799 (38.1)	3086 (39.1)	713 (34.3)
	Mean ± SD	4.8 ± 2.0	4.9 ± 2.0	4.6 ± 2.0

**Figure 1:**
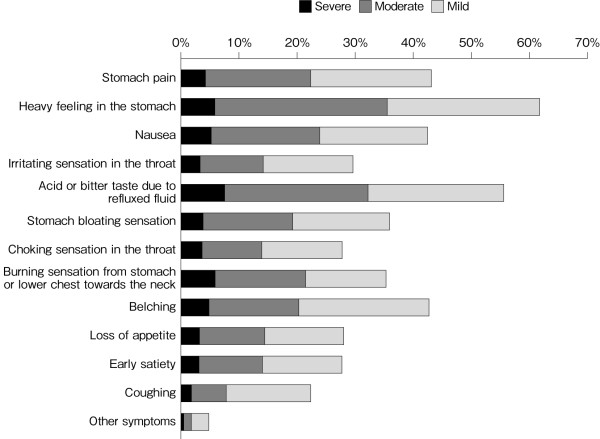
**Profile and intensity of patient-reported symptoms at baseline (N = 8985)**.

Figure 1 summarizes the specific patient-reported symptoms at baseline. "Stomach pain", "heavy feeling in the stomach", "nausea", "acid or bitter taste due to refluxed fluid", and "belching" were reported by ≥40% of patients. Approximately one third of patients complained of a "burning sensation towards the neck" as the typical symptom of heartburn. Three or more symptoms were reported by 75.2% of patients, and six or more symptoms were reported by 31.5% of patients.

**Figure 2:**
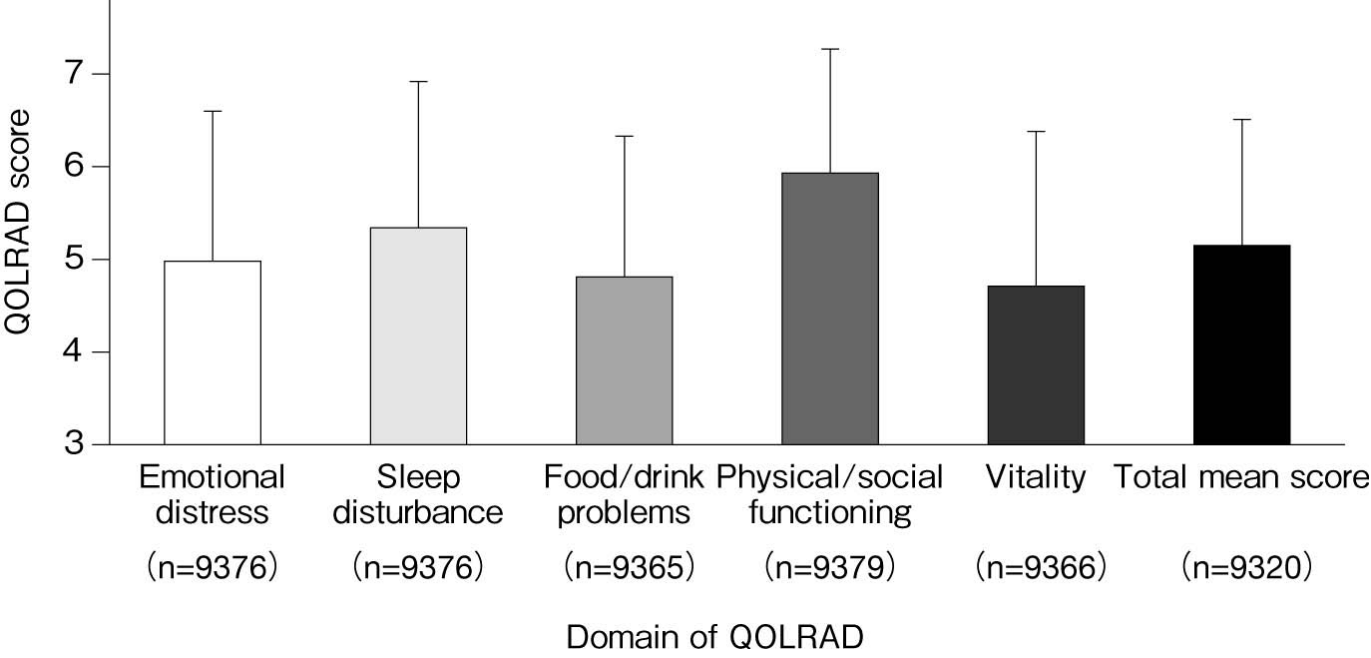
**Mean (± SD) QOLRAD-J domain and total scores at baseline**.

**Figure 3:**
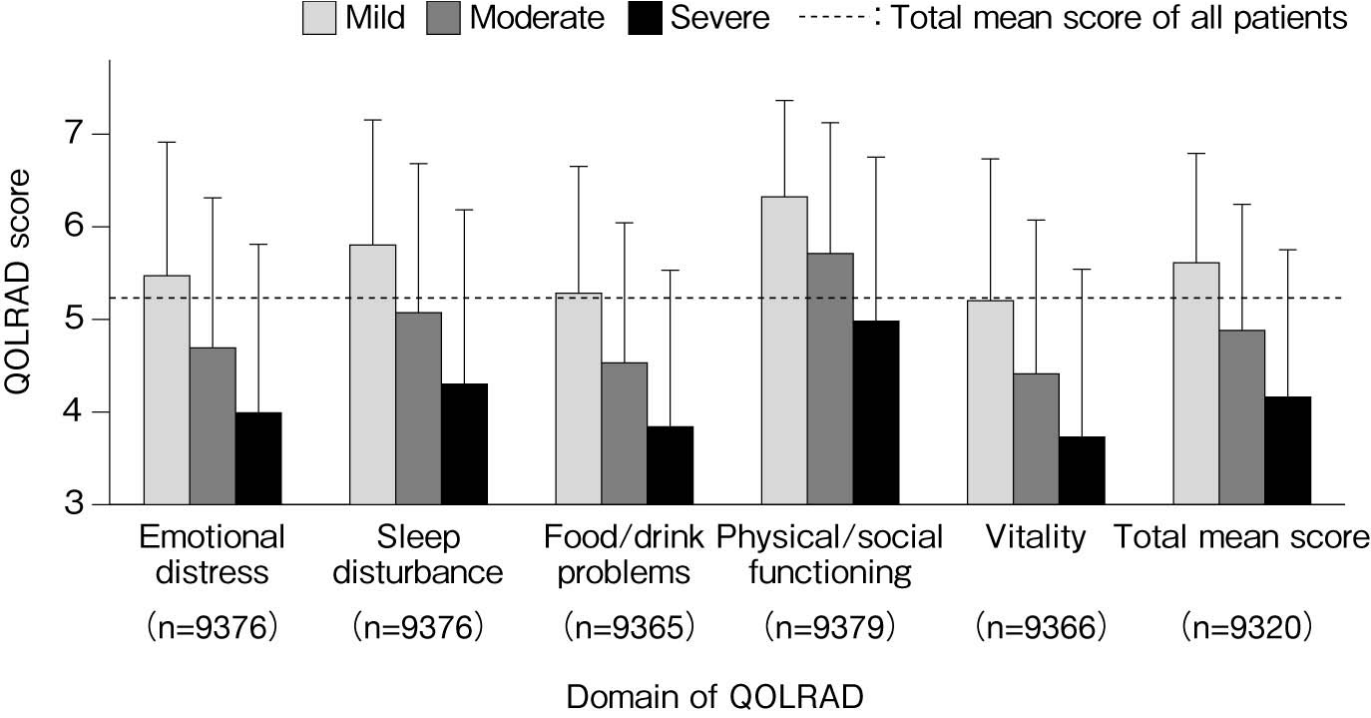
**Mean (± SD) QOLRAD-J domain and total scores according to the intensity of physician-reported symptoms at baseline**.

**Figure 4:**
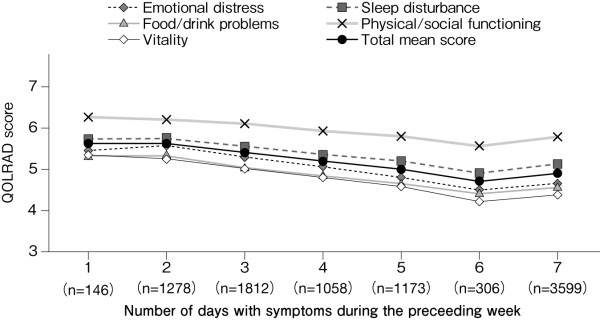
**Mean QOLRAD-J domain and total scores by frequency of symptoms at baseline**.

**Figure 5:**
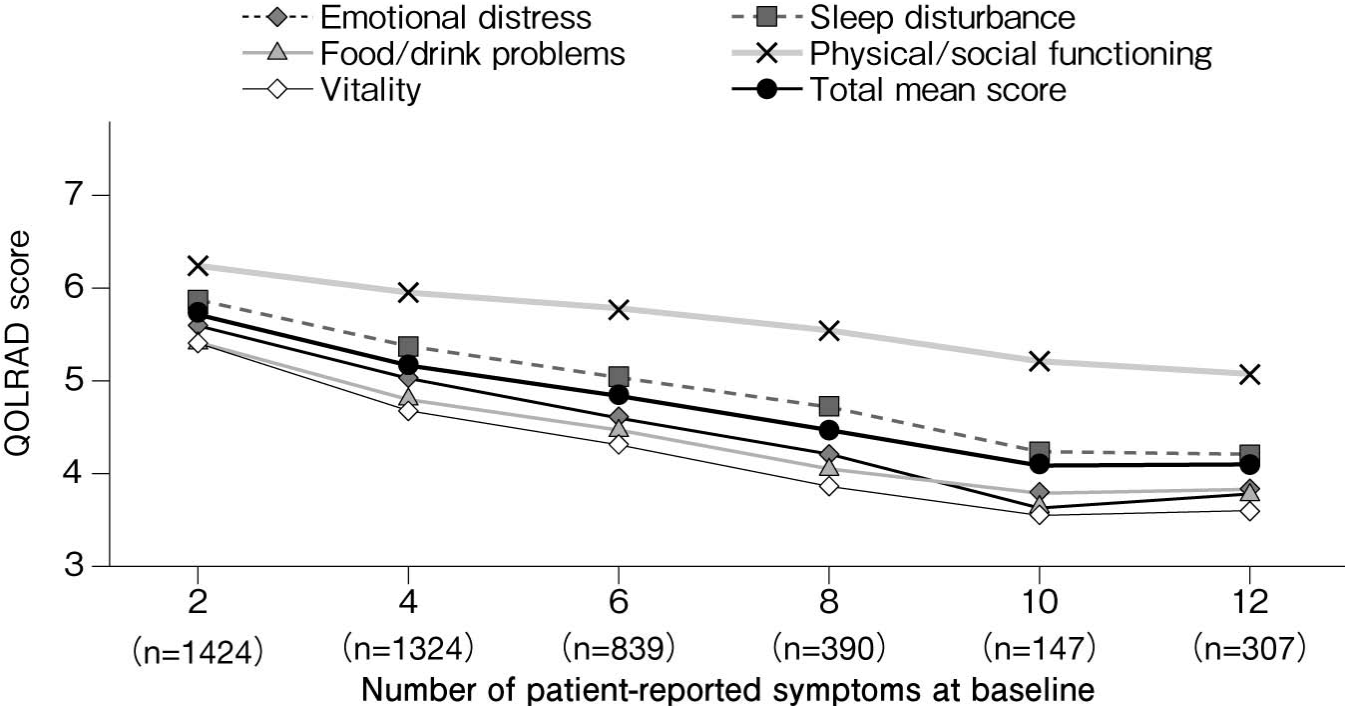
**Mean QOLRAD-J domain and total scores according to the number of patient-reported symptoms at baseline**.

**Figure 6:**
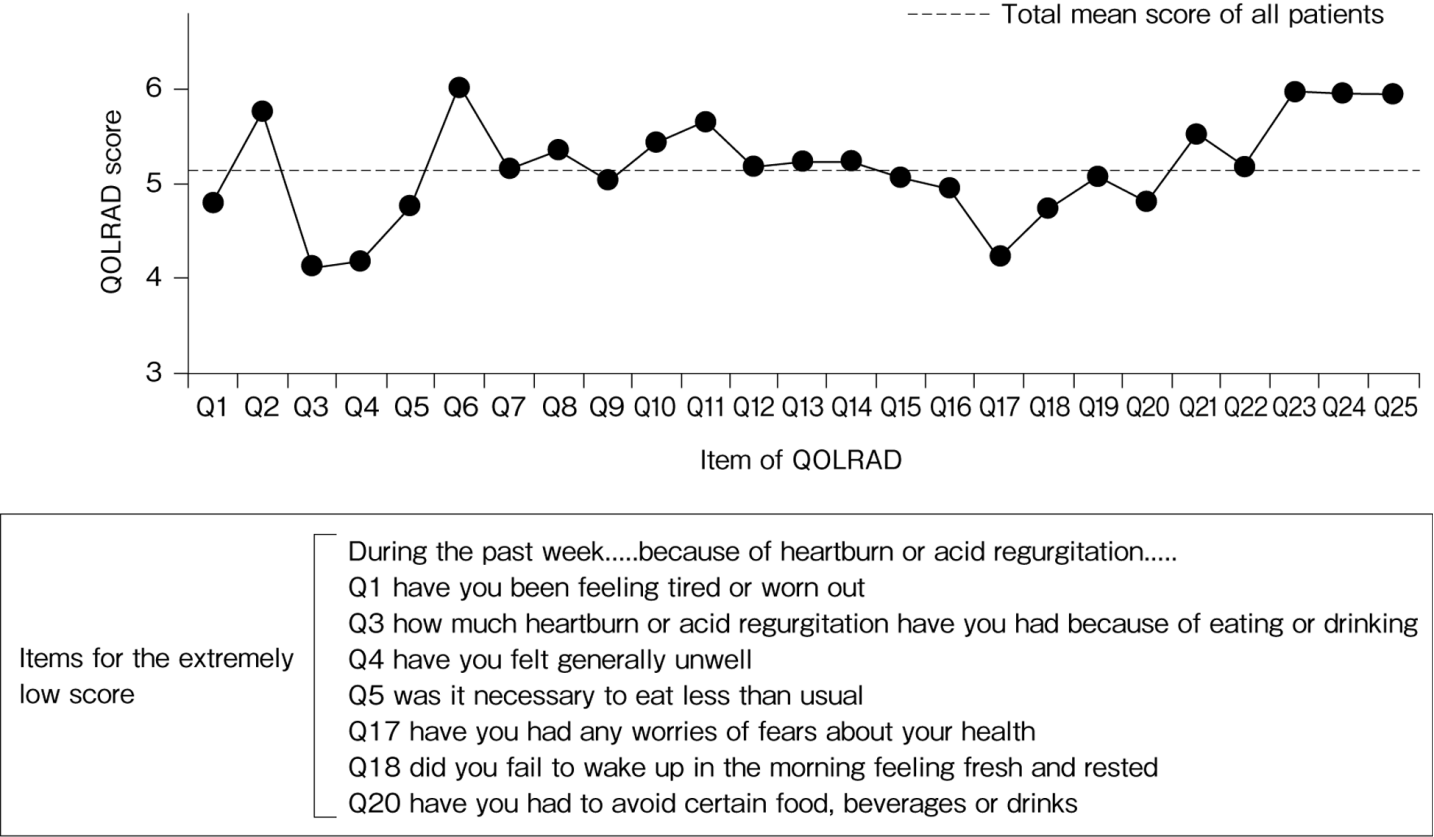
**Mean QOLRAD-J item scores at baseline**.

Figure 2 shows the scores for each domain of the QOLRAD at baseline for all patients. The overall mean score of the omeprazole and comparator groups was similar: 5.14 and 5.22, respectively. Figure 3 shows the scores stratified according to the severity of physician-reported symptoms. The overall mean score (n = 9320) was decreased (5.15), with prominent decreases in the emotional distress, food/drink problems, and vitality domains. The overall mean score and the scores for the individual domains were lower in patients with greater severity, frequency or number of symptoms (Figures 3, 4 and 5). QOLRAD item scores are shown in Figure 6. The Cronbach's α coefficients for individual domains at baseline were 0.93 for emotional distress, 0.91 for sleep disturbance, 0.89 for food/drink problems, 0.90 for physical/social functioning, and 0.82 for vitality.

### Changes in clinical symptoms and QOL during the investigation

#### Change in symptoms

Among patients treated with omeprazole, satisfactory improvement in subjective symptoms was observed in 61.7% and 81.8% of patients at Weeks 4 and 8, respectively, and these percentages were both significantly higher than those of patients treated with other drugs (Table 3). In addition, the percentage of patients with complete resolution of subjective symptoms at Weeks 4 and 8 was 44.0% and 67.2%, respectively, in patients treated with omeprazole, versus 27.3% and 46.0%, respectively, in patients treated with other drugs, showing significant between-group differences at both Weeks 4 and 8 (Table 4).

**Table 3:** Proportion of patients with satisfactory improvement in physician-reported symptoms

	Omeprazole			Other drugs			Omeprazole - Other drugs		
	Improvement, %		95% CI	Improvement, %		95% CI	Difference	95% CI	p-value*
Week 4	61.7	(3265/5291)	(60.4, 63.0)	45.0	(631/1401)	(42.4, 47.6)	16.7	(13.8, 19.6)	< 0.001
Week 8	81.8	(3373/4123)	(80.6, 83.0)	64.2	(697/1085)	(61.4, 67.1)	17.6	(14.5, 20.7)	< 0.001

* χ^2 ^test. CI = confidence interval.

**Table 4:** Complete resolution of physician-reported symptoms

	Omeprazole			Other drugs			Omeprazole - Other drugs		
	Resolution, %		95% CI	Resolution, %		95% CI	Difference	95% CI	p-value*
Week 4	44.0	(2327/5291)	(42.6, 45.3)	27.3	(383/1401)	(25.0, 29.7)	16.6	(14.0, 19.3)	< 0.001
Week 8	67.2	(2771/4123)	(65.8, 68.6)	46.0	(499/1085)	(43.0, 49.0)	21.2	(17.9, 24.5)	< 0.001

* χ^2 ^test. CI = confidence interval.

The rate of improvement of patient-reported symptoms at Week 8 was significantly higher for patients treated with omeprazole than for patients treated with other drugs for symptoms other than "irritating sensation in the throat" and "coughing" (Table 5).

**Table 5:** Proportion of patients showing satisfactory improvement of individual symptoms

	Week 4					Week 8				
		
	Omeprazole Improvement, %	Other drugs Improvement, %	Difference (95% CI)		p-value*	Omeprazole Improvement, %	Other drugs Improvement, %	Difference (95% CI)		p-value*
Stomach pain	71.7 (1107/1544)	64.1 (257/401)	7.6	(2.4, 12.8)	0.003	83.7 (1018/1216)	74.9 (230/307)	8.8	(3.5, 14.1)	< 0.001
Heavy feeling in the stomach	61.7 (1467/2379)	45.9 (299/652)	15.8	(11.5, 20.1)	< 0.001	77.0 (1465/1903)	64.0 (330/516)	13.0	(8.5, 17.6)	< 0.001
Nausea	72.1 (1079/1497)	62.0 (246/397)	10.1	(4.8, 15.4)	< 0.001	85.1 (1013/1191)	74.2 (227/306)	10.9	(5.6, 16.2)	< 0.001
Irritating sensation in the throat	68.4 (726/1061)	69.1 (170/246)	-0.7	(-7.1, 5.7)	0.836	77.8 (657/844)	71.4 (135/189)	6.4	(-0.6, 13.4)	0.059
Acid or bitter taste due to refluxed fluid	70.7 (1502/2124)	58.4 (320/548)	12.3	(7.8, 16.9)	< 0.001	85.4 (1492/1747)	72.9 (315/432)	12.5	(8.0, 17.0)	< 0.001
Stomach bloating sensation	66.4 (878/1322)	56.7 (202/356)	9.7	(3.9, 15.4)	< 0.001	81.5 (882/1082)	74.0 (211/285)	7.5	(1.9, 13.1)	0.005
Choking sensation in the throat	66.8 (641/959)	64.7 (152/235)	2.2	(-4.6, 9.0)	0.530	78.6 (611/777)	68.9 (126/183)	9.8	(2.5, 17.1)	0.005
Burning sensation from stomach or lower chest toward the neck	78.9 (1097/1391)	69.2 (225/325)	9.6	(4.2, 15.1)	< 0.001	88.4 (989/1119)	77.0 (207/269)	11.4	(6.1, 16.8)	< 0.001
Belching	60.7 (940/1548)	49.3 (197/400)	11.5	(6.0, 16.9)	< 0.001	74.3 (947/1275)	64.4 (206/320)	9.9	(4.1, 15.7)	< 0.001
Loss of appetite	75.4 (771/1022)	66.2 (178/269)	9.3	(3.0, 15.5)	0.002	85.3 (721/845)	75.1 (154/205)	10.2	(3.8, 16.6)	< 0.001
Early satiety	68.8 (698/1015)	62.0 (178/287)	6.7	(0.5, 13.0)	0.031	80.5 (656/815)	72.0 (167/232)	8.5	(2.1, 14.9)	0.005
Coughing	62.9 (450/715)	58.9 (103/175)	4.1	(-4.0, 12.2)	0.319	74.6 (432/579)	68.7 (101/147)	5.9	(-2.4, 14.2)	0.148

* χ^2 ^test. CI = confidence interval.

#### Change in QOL

**Figure 7:**
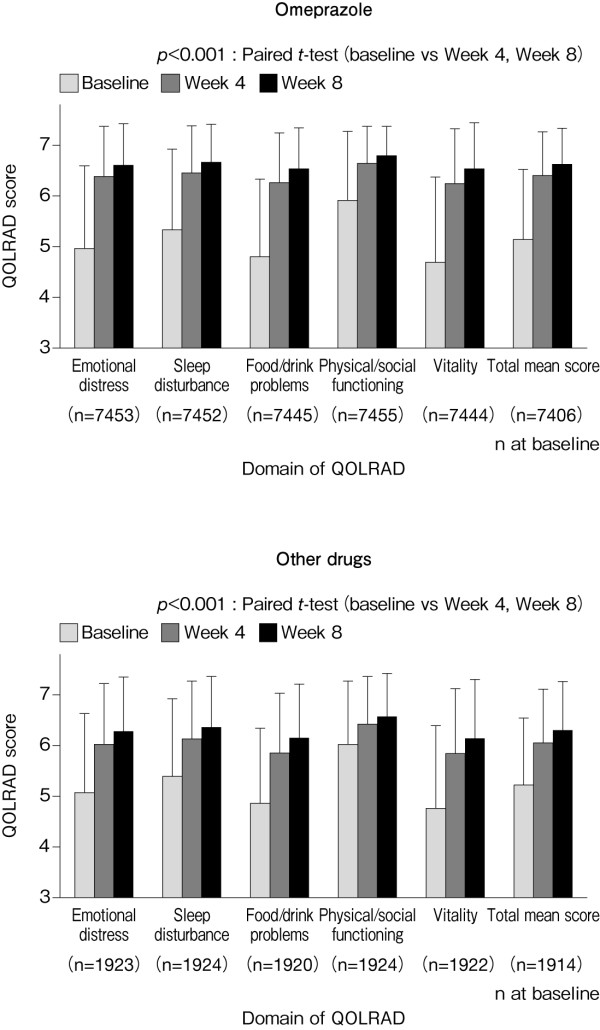
**Mean (± SD) QOLRAD-J domain and total scores at baseline, and at Weeks 4 and 8**.

**Figure 8:**
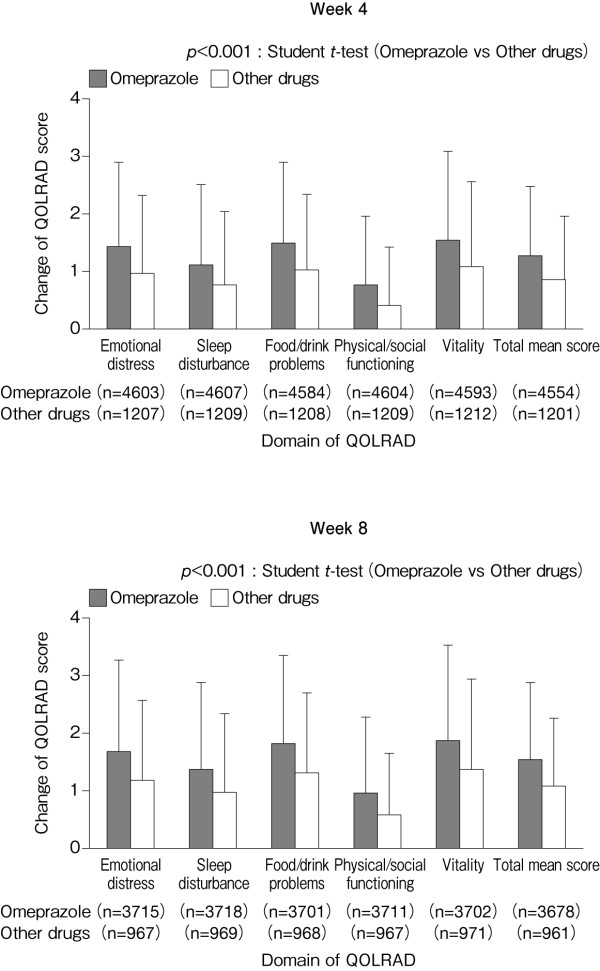
**Mean (± SD) change in QOLRAD-J domain and total scores from baseline to Weeks 4 and 8**.

In both the omeprazole and other drugs group, the overall mean QOLRAD score and the QOLRAD scores for individual domains at Week 4 improved significantly from baseline (Figure 7). However, the degree of improvement (change) was significantly greater in the omeprazole group than in the other drugs group (Figure 8).

### Safety and tolerability

Adverse drug reactions (adverse events related to omeprazole) are summarized in Table 6. Overall, 0.92% of patients experienced adverse drug reactions, with no differences in their incidence among different doses. The most common adverse drug reactions were gastrointestinal disorders (0.35%) followed by skin and subcutaneous tissue disorders (0.22%). Four serious adverse drug reactions, namely intracranial venous sinus thrombosis, vertigo, upper abdominal pain, and increased blood pressure, were reported in one patient each.

**Table 6:** Rate of adverse drug reaction by daily dose^1)^

		Daily dose of omeprazole		
	Total (N = 7711)	10 mg (N = 2956)	20 mg (N = 4726)	40 mg^2)^ (N = 29)
No. of patients with adverse drug reactions	71	26	45	0
No. of adverse drug reactions	73	28	45	0
Rate of adverse drug reactions	0.92%	0.88%	0.95%	0%

**Preferred term^3)^**				
Diarrhoea	7 (0.09)	3 (0.10)	4 (0.08)	0
Abdominal distension	5 (0.06)	2 (0.07)	3 (0.06)	0
Drug eruption	5 (0.06)	1 (0.03)	4 (0.08)	0
Thirst	5 (0.06)	1 (0.03)	4 (0.08)	0
Pruritus	4 (0.05)	2 (0.07)	2 (0.04)	0
Upper abdominal pain	3 (0.04)	0	3 (0.06)	0
Constipation	3 (0.04)	1 (0.03)	2 (0.04)	0
Vomiting	3 (0.04)	1 (0.03)	2 (0.04)	0
Eczema	3 (0.04)	1 (0.03)	2 (0.04)	0
Rash	3 (0.04)	1 (0.03)	2 (0.04)	0
Abnormal hepatic function	2 (0.03)	0	2 (0.04)	0
Muscle pains	2 (0.03)	1 (0.03)	1 (0.02)	0

Values are n (%).

^1) ^Some patients have more than one adverse drug reaction; events occurred in two or more patients are listed.

^2) ^20 mg twice daily.

^3) ^MedDRA Ver12.0.

## Discussion

This is the first large-scale observational investigation that prospectively assessed the changes in clinical symptoms and QOL over 8 weeks in Japanese RE patients treated with omeprazole.

The QOL questionnaire used in this investigation, the QOLRAD-J, was validated and found to be useful in 224 Japanese patients with heartburn[9]. In the present investigation, the Cronbach's α coefficients were calculated for the individual domains of the QOLRAD at baseline to verify the reliability of the questionnaire in patients with RE. Notably, the coefficients were quite high (0.82-0.93) and were similar to those reported by Hongo et al. (0.83-0.94)[9], supporting the internal consistency within each domain. In addition, the severity of physician-reported symptoms showed a negative correlation with QOLRAD scores. Taken together, the QOLRAD-J was shown to be a reliable questionnaire to assess QOL in Japanese patients with RE.

In an European study that followed 152 patients with RE for 10 years and compared the QOL assessed using the SF-36 between RE patients and the general population, physical function and social function were reported to be significantly lower in patients with RE[12]. According to a report by Talley et al.,[13] the smallest clinically meaningful change in score on the QOLRAD is 0.5, with 1.0 regarded as an 'important change' and 1.5 as a 'very important change'. In the present investigation, the QOLRAD-J score at baseline was found to be lower than the best possible score (7) by 1.5 or more for all domains except physical/social functioning, and low QOL scores were associated with the type, frequency, severity, and number of symptoms. To date, lesion healing has been the focus of treatment for RE; however, the treatment of symptoms is also important in terms of QOL improvement.

A close correlation has been found between QOL and RE symptoms, and these patients also present with a range of other symptoms. Adachi et al. reported that acid reflux symptoms such as heartburn were present in approximately 70% of patients with RE, concurrently with stomach pain in 54% and a "heavy feeling in the stomach" in 62%[14]. In the present investigation, a "heavy feeling in the stomach" was the most commonly reported symptom and its severity was higher than that of other symptoms. In addition, ≥70% of the patients had three or more symptoms. These results were consistent with those reported by Adachi et al.,[14] demonstrating that patients with RE suffer not only typical symptoms such as heartburn but also various other symptoms, confirming the importance of evaluating those symptoms during treatment. The baseline characteristics of patients in our study were similar to those in the earlier study [14], although fewer patients with BMI >25 participated in our study (18% *versus *30%). It is possible that the nature and severity of symptoms might vary among patient groups which differ significantly with respect to baseline characteristics, from those studied to date.

Omeprazole was more efficacious than other drugs in improving RE symptoms in the present study. The efficacy of PPIs in the treatment of upper gastrointestinal symptoms has been demonstrated in Japanese and non-Japanese studies. For example, in the CADET-HN study, patients with unexamined dyspepsia symptoms were treated with omeprazole, H_2 _blockers, prokinetics or placebo, and the results showed that omeprazole was significantly more effective in improving symptoms[15]. Similarly, in the Japanese J-FOCUS study conducted in a similar patient population, omeprazole elicited significantly greater improvements in symptoms compared with H_2 _blockers, prokinetics, and gastric mucoprotective drugs[16]. The present investigation further supports the results of these previous studies demonstrating the usefulness of PPIs such as omeprazole in the treatment of upper abdominal symptoms.

At Week 8, patients treated with other drugs did not show improvements in any QOLRAD domain by a score of 1.5 or more (i.e., a 'very important change' as described by Talley et al.[13]). By contrast, patients treated with omeprazole showed improvements in scores of 1.5 or more for the domains of emotional distress, food/drink problems, and vitality, indicating the favourable efficacy of omeprazole in improving symptoms and, in turn, QOL. Havelund et al.[17] compared changes in QOL assessed using GSRS and PGWB after 4 weeks of treatment with omeprazole (20 or 10 mg/day) or placebo in patients with heartburn but without esophagitis. Both omeprazole groups showed significantly greater improvements in QOL than the placebo group, and the PGWB scores in the two omeprazole groups improved to a level comparable with that of healthy individuals. In addition, omeprazole elicited marked improvements in the domains of anxiety, depression, and self-control[17]. In the present investigation, there were marked improvements in the QOLRAD domains of emotional distress and vitality, indicating that omeprazole can improve the psychological aspects of QOL in patients with RE.

## Conclusions

In this large-scale survey, omeprazole improved symptoms and QOL more effectively in Japanese patients with RE than other investigated drugs, and had a good tolerability profile.

## Abbreviations

GERD: gastroesophageal reflux disease; PGWB: Psychological General Well-Being; PPI: proton pump inhibitor; QOL: quality of life; QOLRAD: Quality of Life in Reflux and Dyspepsia; RE: reflux esophagitis.

## Declaration of Competing interest

All authors are employees of AstraZeneca K.K., the manufacturer of omeprazole.

## Authors' contributions

SY contributed to study concept, study design, and writing manuscript. MN performed statistical analyses and contributed to writing manuscript. MD contributed to data interpretation and writing manuscript. All authors read and approved the final manuscript.

## Pre-publication history

The pre-publication history for this paper can be accessed here:

http://www.biomedcentral.com/1471-230X/11/15/prepub
